# Detection of anti-*Mycoplasma bovis* IgG in bovine nasopharyngeal swabs

**DOI:** 10.3389/fvets.2026.1856081

**Published:** 2026-06-11

**Authors:** Ethan P. Dudley, Brennen O. Hunt, Robert Valeris-Chacin

**Affiliations:** 1 Department of Veterinary Pathobiology, College of Veterinary Medicine and Biomedical Sciences, Texas A&M University, Canyon, TX, United States; 2 Department of Large Animal Clinical Sciences, College of Veterinary Medicine and Biomedical Sciences, Texas A&M University, Canyon, TX, United States

**Keywords:** cattle, IgG, mucosal immunity, *Mycoplasma bovis*, respiratory tract

## Abstract

*Mycoplasma bovis* is a significant bacterial pathogen involved in the Bovine Respiratory Disease (BRD) complex. While assessing localized mucosal immunity is vital for understanding host defenses against respiratory pathogens, there is a lack of validated diagnostic assays for detecting mucosal immunoglobulins. This study aimed to evaluate the diagnostic performance of a novel multiplex ELISA platform (Pictor PictVet^®^) in detecting anti-*M. bovis* IgG in bovine nasopharyngeal swab washes. Archived nasopharyngeal swab washes from crossbred feedlot steers (50 positive and 50 negative for *M. bovis* DNA via digital qPCR) were evaluated. The ELISA utilized three target antigens: K-0310, K-0320, and MilA. Diagnostic accuracy was assessed using both concurrent *M. bovis* DNA status and a Latent Class Analysis (LCA) model as ground truths. Inter-operator reproducibility and analytical sensitivity (by serial dilution) were also determined. The MilA antigen exhibited high variance and poor predictive performance (51.5% accuracy), resulting in its exclusion from the final models. When utilizing *M. bovis* DNA status as the ground truth, the best custom cutoff (K-0310 and K-0320 in series) yielded high specificity (88%) but low sensitivity (30%). However, employing LCA to estimate IgG status (17.1% prevalence) dramatically improved diagnostic validity. Evaluating K-0310 and K-0320 in parallel achieved an accuracy of 97.59% with 100% sensitivity and 95.18% specificity. The assay demonstrated excellent inter-operator agreement (Kappa = 0.894 to 1) and reliably detected positive samples at a 1:2 dilution. Analyzing nasopharyngeal swabs with this novel multiplex ELISA platform, specifically utilizing the K-0310 and K-0320 antigens, provides a highly accurate, reproducible, and non-invasive method for monitoring mucosal anti-*M. bovis* IgG. The use of LCA was paramount in establishing appropriate cutoffs, highlighting the limitations of relying solely on pathogen DNA detection to evaluate adaptive immune responses.

## Introduction

Bovine respiratory disease (BRD) represents one of the most persistent and economically devastating health challenges in the global cattle industry. The pathogenesis of BRD is multifactorial, typically characterized by primary viral infections followed by secondary bacterial proliferation (1). The bacterial agents implicated in the BRD complex include *Mannheimia haemolytica, Histophilus somni, Pasteurella multocida*, and *Mycoplasma bovis* (2). Among these, *M. bovis* is particularly challenging due to its association with chronic, unresponsive pneumonia and systemic manifestations (3–6), which complicate management and treatment strategies.

The immune response to *M. bovis* in the bovine respiratory tract involves a complex interplay of cellular and humoral mechanisms that, despite their intensity, often fails to completely clear the pathogen. Upon colonizing the respiratory mucosa, *M. bovis* triggers a localized inflammatory response characterized by the recruitment of innate immune cells, such as macrophages and neutrophils, alongside a marked hyperplasia of the bronchus-associated lymphoid tissue (BALT) (4, 7). The adaptive immune arm is also activated, including local T-cells and B-cells, leading to an expansion of plasma cells that secrete *M. bovis*-specific immunoglobulins, predominantly IgG (particularly the IgG1 subclass) and IgA, into the mucosal environment (8, 9). However, *M. bovis* is highly adept at immune evasion. Several virulence factors have been implicated in the ability of *M. bovis* to persist in the lungs of cattle with BRD, such as variable surface proteins (Vsp), adhesins, nucleases, H_2_O_2_ production, and biofilm formation (10, 11).

Assessing the bovine immune response to *M. bovis* is critical for developing effective preventative and therapeutic interventions. While systemic humoral immunity has been the focus of traditional serological diagnostics, the localized mucosal immunity of the respiratory tract acts as the primary frontline defense against colonization (9). Monitoring specific mucosal immunoglobulins, notably IgG, can yield important insights into the localized host-pathogen dynamics and the local immune response. Additionally, bovine nasopharyngeal swabs represent a practical and minimally invasive alternative for assessing the upper respiratory tract environment (12).

While highly sensitive molecular techniques, such as quantitative PCR (qPCR), are routinely utilized to quantify *M. bovis* DNA and confirm the pathogen's presence in biological samples, DNA detection alone does not characterize the host's concurrent local immune status (13). Currently, there is a distinct lack of rigorously validated diagnostic assays specifically designed to detect localized anti-*M. bovis* antibodies in mucosal swab. Therefore, the objective of this study was to evaluate the diagnostic performance of the novel Pictor PicVet^®^ *Mycoplasma bovis* IgG multiplex enzyme-linked immunosorbent assay (ELISA) platform utilizing specific target antigens (K-0310, K-0320, and MilA) for the detection of anti-*M. bovis* IgG (14), in bovine nasopharyngeal swabs. Specifically, we aimed to assess the assay's accuracy, inter-operator reproducibility, and analytical sensitivity.

## Materials and methods

### Description of samples

For this study, we used archived nasopharyngeal swabs collected in a prior study evaluating the effect of antimicrobial metaphylaxis for BRD on the host transcriptome response (15). In brief, nasopharyngeal swabs were collected from 105 crossbreed steers on day 0, 3, 7, 14, 21, and 56 post arrival at the feedlot (*n* = 621). All experimental protocols were approved by the West Texas A&M University Institutional Animal Care and Use Committee (2022.03.002). All methods were carried out in accordance with relevant guidelines and regulations, including obtaining informed consent from the owner for animal enrollment in the research. A complete description of the experimental design was reported using the ARRIVE guidelines in Bigelow et al. (15).

### Quantification of *Mycoplasma bovis* DNA

Cuts of the nasopharyngeal swabs (approximately 250 mg) were used for DNA extraction using the QIAamp PowerSoil Fecal Pro DNA Extraction Kit (QIAGEN Inc., Germany) on a QIAcube Connect device (QIAGEN Inc., Germany) and stored at −80°C. Extracted DNA was employed in the detection of *M. bovis* via a microfluidic digital qPCR (QuantStudio Absolute Q, Applied Biosystems, Waltham, MA, USA) in a multiplex assay, detecting *M. bovis, Mannheimia haemolytica, Histophilus somni*, and *Pasteurella multocida*. We used primers and TaqMan probes described elsewhere (Loy et al., 2018; Goecke et al., 2021), except all quenchers were modified to QSY, and the dyes were selected to match the QuantStudio Absolute digital PCR capabilities (see Supplementary Materials for a complete list).

The final digital qPCR reaction (9 μL) contained 40 ng of sample DNA, PCR Master Mix (1X), the primers (forward and reverse, both at 400 nM), and probes (400 nM), with molecular water to complete the final reaction volume. The PCR reaction volume was transferred to the MAP16 plate and overlaid with the isolation buffer. The thermocycling conditions were: hot start at 96°C for 10 min and 40 cycles of 96°C for 5 s and 60°C for 15 s. Molecular-grade water was used as the negative template control, and DNA extractions from ATCC strains were used as the positive control (see Supplementary Materials for the complete list). Concentration of the dqPCR targets was reported as genome equivalents/40 ng of sample DNA.

### Selection of samples

Bovine nasopharyngeal swabs (*n* = 100) with known *M. bovis* DNA status (50 positive and 50 negative for the presence of *M. bovis* DNA via digital qPCR) were selected for this study. The *M. bovis* DNA negative samples were confirmed as negative across three independent runs. *Mycoplasma bovis* DNA concentration ranged from 444.7 to 9,317.2 *M. bovis* genome equivalents/40 ng of sample DNA. The swabs were cut (approximately 250 mg) and maintained at −80°C in Eppendorf tubes until further processing. Swab cuts were washed with PBS (1 mL) and incubated overnight at 4°C. After incubation, the wash was recovered and transferred to a new Eppendorf tube. Of note, the recovered volume was variable among the samples.

### Accuracy assessment

For accuracy assessment, samples were randomized to plates and wells. The ELISA procedure was followed as described elsewhere (14). The only modification was that 50 μL of sample (swab wash) was added per well in duplicates. This volume was determined in a pilot experiment comparing the average median intensity for each antigen between two different sample volumes (50 and 200 μL). In brief, six samples were selected for this pilot, two with high *M. bovis* DNA concentration, two with medium-low *M. bovis* DNA concentration, and two *M. bovis* DNA-negative samples. The ELISA procedure was performed on 50 and 200 μL of swab wash from each sample in duplicates. It was determined that 50μL allowed for a similar characterization to using 200μL. Additionally, positive and negative controls were added to each plate in duplicates.

### Reproducibility assessment

Five composite samples from samples with similar average median intensities were created for the reproducibility assessment. Three composite samples were expected to be positive (using one or both cutoffs), and two were expected to be negative for both cutoffs (see section 2.7). The reproducibility experiment involved performing the ELISA on the five composite samples using two kits, over two days, by two operators. Composite samples, positive and negative controls, were added to each plate in duplicates. No deviations from the protocol were made except for the sample volume, which was 50μL as in section 2.4.

### Analytical sensitivity

Swab wash was used for this experiment as indicated in the protocol (100μL of sample volume per well). Ten samples classified as positive with both cutoff schemes (see 2.7 section) and with sufficient volume for this experiment (a minimum of 400μL) were selected. Undiluted samples (swab wash) were added to the starting wells in duplicate (200μL per well). Then, a 2-fold serial dilution was performed in sterile PBS until a 1:128 dilution was obtained. Positive and negative controls were added to each plate in duplicates. The ELISA procedure was performed as indicated in the Pictor PictVet^®^ *Mycoplasma bovis* IgG Multiplex ELISA protocol without modifications.

### Statistical analysis

Data were analyzed using Stata/MP 19.5. For the accuracy assessment experiment, values between duplicates for each antigen were averaged (per sample). Logistic regression models were built, regressing the *M. bovis* DNA status on the average median intensity (one model per antigen). A Receiver Operating Characteristic (ROC) curve was created to estimate the area under the curve (an estimator of accuracy). Different cutoff schemes were evaluated: default cutoff (fitted values ≥ 0.5) in parallel and in series, and a custom cutoff with the fitted values with the highest accuracy per antigen in parallel and in series. Additionally, a Latent Class Analysis (LCA) model (with two classes) was built using the average median intensity of the three antigens as outcome (16). The fitted values from this model were dichotomized at the 0.5 threshold to generate the new ground truth variable. The logistic regression models were repeated with this new outcome, and new cutoff values were generated: only the custom cutoffs based on the fitted values with the highest accuracy per antigen in parallel and in series, were evaluated.

For the reproducibility assessment, the values between duplicates for each antigen (per sample per plate) were averaged, and the antigen median intensity variability due to operators and run order (1st or 2nd run of the day) was estimated via the intraclass correlation coefficient (ICC) (17) and visualized using Bland-Altman plots. The final cutoff schemes obtained during the accuracy assessment data analysis were applied to classify each sample, that is, the *M. bovis* DNA-based cutoff (average median intensity ≥ 18.75 for K-0310 and ≥17.5 for K-0320) and the LCA-based cutoff (average median intensity ≥ 31.25 for K-0310 or ≥36 for K-0320). Agreement between operators by antigen (adjusting for day and run order) was estimated using the kappa coefficient and the Gwet's AC1 (18). Additionally, the coefficient of variation was calculated by sample ID and antigen.

For the analytical sensitivity determination, the final cutoff schemes were used to classify each sample:replicate:dilution combination. A Generalized Estimating Equation model, assuming a binomial distribution, logit link function, independent working correlation, and robust standard errors, was fitted to each cutoff with the log_2_(dilution factor) as the predictor. Fitted values were calculated to estimate the probability of classifying the samples as positive depending on the cutoff and the dilution factor.

## Results

### Accuracy assessment

When assessing the performance of the Pictor PictVet^®^ *Mycoplasma bovis* IgG multiplex ELISA, four instances (out of 928) of spot variance exceeding tolerance (as determined using the Pictorial^®^ version 1.3.0 image analysis software) were detected for the antigen MilA. The positive control average median intensity values were >30, except for MilA, which ranged from 14.5 to 24.5. The average median intensity values for all antigens were 0 in the negative control and blank wells.

Using the *M. bovis* DNA status as ground truth, poor predictive performance was observed for MilA (accuracy of 51.5%, Se=60%, Sp=38%). Therefore, it was not used to create the *M. bovis* DNA-based cutoffs. Out of the cutoffs evaluated, the custom cutoffs with the fitted values with the highest accuracy per antigen in series (corresponding to average median intensity ≥ 18.75 for K-0310 and ≥17.5 for K-0320) performed the best (accuracy of 59%, Se=30%, Sp=88%, Supplementary Figure 1).

**Figure 1:**
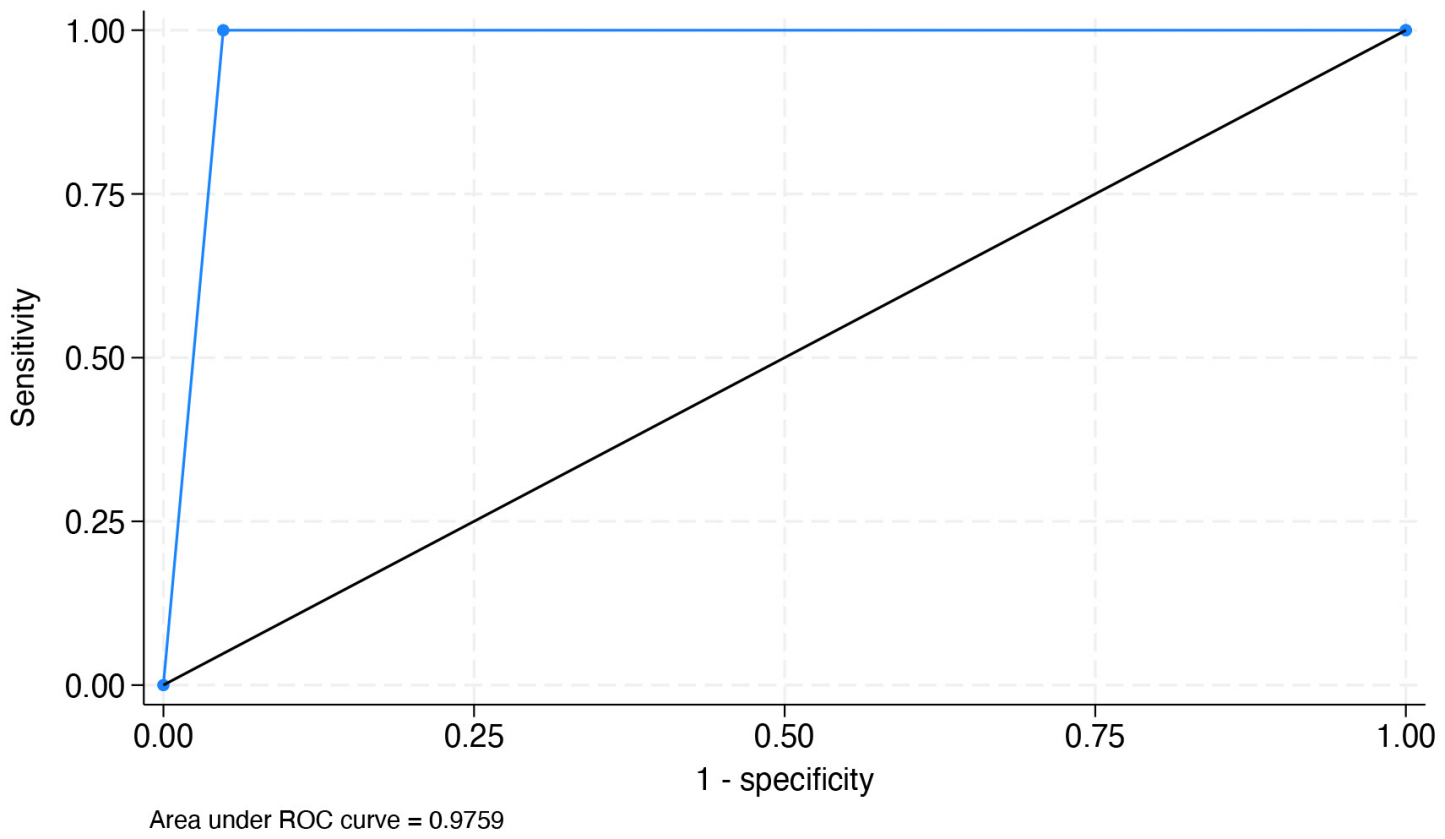
Receiver operating characteristic (ROC) curve for latent class analysis-based cutoffs. The cutoffs correspond to an average median intensity of 31.25 for K-0310 or 36 for K-0320.

As the validity was low, an attempt to estimate the two theoretical subpopulations of samples (*M. bovis* IgG positive and negative samples) was made via LCA. In this model, MilA was again non-informative and was dropped from a subsequent model. The LCA model with only K-0310 and K-0320 estimated that 17.1% of the samples were *M. bovis* IgG positive. Using that estimated membership, the best cutoffs were obtained using the custom cutoff with the fitted values with the highest accuracy per antigen in parallel (average median intensity ≥ 31.25 for K-0310 or ≥36 for K-0320), with an accuracy of 97.59%, a Se = 100%, and a Sp = 95.18% (Figure 1). Of note, using only K-0320 (same cutoff) also results in high validity (with perfect Sp, but Se = 76.47%).

### Reproducibility assessment

When assessing the reproducibility, eleven instances (out of 512) of spot variance exceeding tolerance were detected for the antigen MilA and one for K-0320 in the composite samples. The positive control average median intensity values were >30, except for MilA, which ranged from 23.5 to 29 in six instances. The average median intensity values for all antigens were < 8 in the negative control, except for two instances where the values were 14.5 and 16, respectively, for K-0320. The average median intensity values for all antigens were 0 in blank wells.

In general, very low variability was observed in the values of antigen median intensity between the two operators (Table 1). The ICC values were lower, indicating more variability, for MilA. Some of those coefficients had wide 95% confidence intervals (CI), suggesting caution should be exercised when interpreting them. Similarly, very low variability was observed between runs of the same day, with the exception of MilA, where values were lower with very wide 95% CI (Table 2). It is important to highlight that run order and kit variables were collinear.

**Table 1:** Antigen median intensity variability due to operators.

Antigen	Day	Order	ICC	95% CI
K-0310	1	1	0.979	0.832, 0.998
		2	0.997	0.935, 1
	2	1	0.968	0.19, 0.997
		2	0.993	0.616, 0.999
K-0320	1	1	0.946	0.646, 0.994
		2	0.994	0.852, 0.999
	2	1	0.968	0.082, 0.997
		2	0.981	0.539, 0.998
MilA	1	1	0.888	0.396, 0.987
		2	0.843	−0.049, 0.983
	2	1	0.884	0.316, 0.987
		2	0.955	0.702, 0.995

ICC, intraclass correlation coefficient; a value of 1 in ICC indicates perfect agreement.

**Table 2:** Antigen median intensity variability due to the order of run.

Antigen	Day	Operator	ICC	95% CI
K-0310	1	A	0.98	0.823, 0.998
		B	0.95	0.604, 0.995
	2	A	0.979	0.814, 0.998
		B	0.992	0.928, 0.999
K-0320	1	A	0.931	0.489, 0.993
		B	0.965	0.707, 0.996
	2	A	0.98	0.826, 0.998
		B	0.989	0.898, 0.999
MilA	1	A	0.627	−0.376, 0.953
		B	0.86	0.162, 0.984
	2	A	0.807	−0.012, 0.978
		B	0.806	−0.016, 0.978

ICC, intraclass correlation coefficient; a value of 1 in ICC indicates perfect agreement.

**Figure 2:**
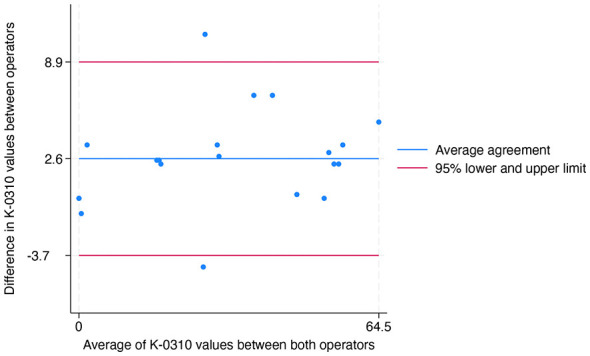
Bland-Altman plot for K-0310. The average K-0310 intensity values for both operators are plotted against the difference between their K-0310 values. The blue horizontal line is the average agreement, and the red horizontal lines are the 95% lower and upper limits.

**Figure 3:**
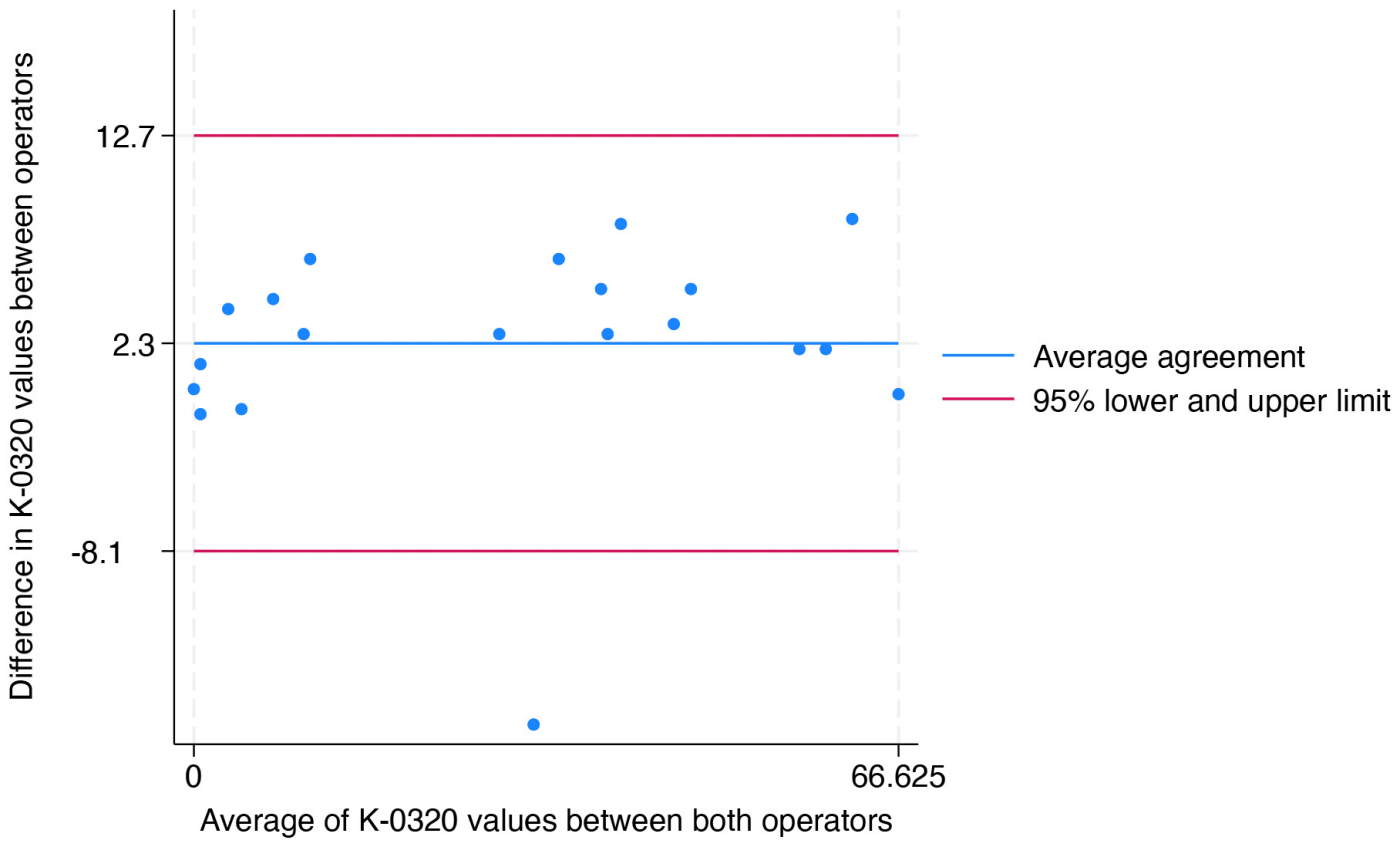
Bland-Altman plot for K-0320. The average K-0320 intensity values for both operators are plotted against the difference between their K-0320 values. The blue horizontal line is the average agreement, and the red horizontal lines are the 95% lower and upper limits.

Systematic differences between the operators are depicted in Figures 2, 3, and Supplementary Figure 2. Operator A obtained systematically higher values for all three antigens than operator B. This behavior was more accentuated for K-0320.

The coefficient of variation (CV) for K-0310 ranged from 0.1 to 0.21 (that is, 10 to 21%) in the positive samples and 0.17 to 2 in the negative samples (as a result of the high number of zeros). For K-0320, the CV ranged from 0.06 to 0.28 in positive samples, and from 0.28 to 1.53 in the negative samples. By contrast, the CV for MilA ranged from 0.21 to 0.7 in positive samples and from 0.12 to 0.93 in negative samples.

Excellent agreement between the operators was observed when the *M. bovis* DNA-based cutoffs were applied. The kappa coefficient was 1 and the Gwet's AC1 was 1. The probabilistic benchmark intervals could not be estimated. When the LCA-based cutoffs were applied, the kappa coefficient was 0.894 and the Gwet's AC1 was 0.906, both in the probability benchmark interval of 0.6–0.8 (substantial agreement).

### Analytical sensitivity

When assessing analytical sensitivity, one instance of spot variance exceeding tolerance was detected for the antigen MilA. The positive control average median intensity values were >30 except for MilA, where they ranged from 14 to 24.5. The average median intensity values for all antigens were 0 in the negative control and blank wells.

**Figure 4:**
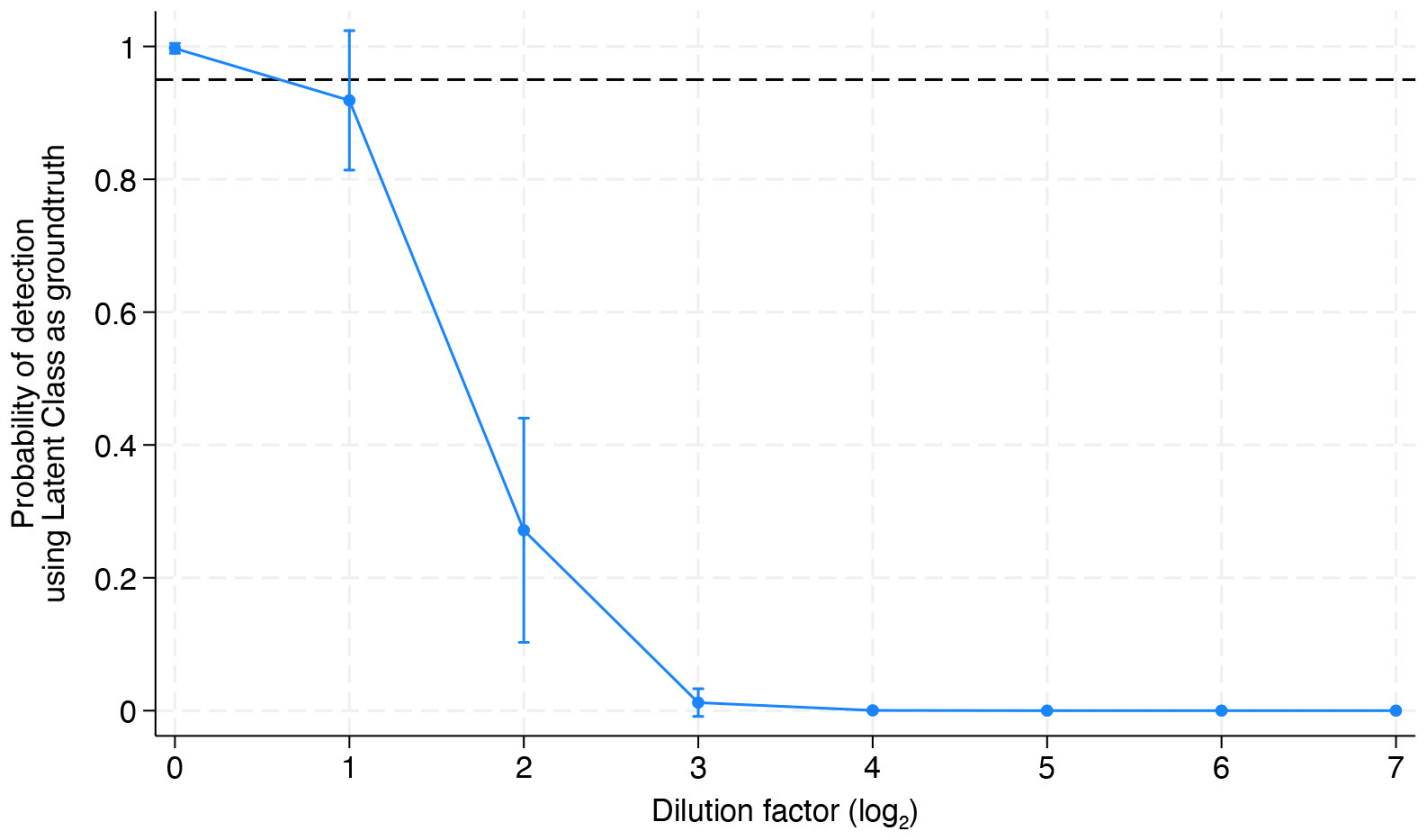
Limit of detection using a latent class analysis-based cutoff. The y-axis represents the probability of detection using a Latent Class Analysis-based cutoff, and the x-axis depicts the dilution factor (transformed as log_2_). Vertical lines represent 95% CI adjusted for clustering. The horizontal dashed line marks the target probability of 0.95.

Considering the *M. bovis* DNA-based cutoff, the probability associated with the dilution factor 2 (that is, 1:2) was 0.853 (or 85.3%) with 95% CI: 0.725–0.98 (or 72.5%−98%), including the target probability for this experiment (0.95 or 95%, Supplementary Figure 3). A similar result was observed with the LCA-based cutoff. The probability associated with the dilution factor 2 was 0.919 (95% CI: 0.814–1; Figure 4).

## Discussion

Accurate assessment of mucosal immunity is crucial for understanding the host response to respiratory pathogens such as *M. bovis*. In this study, we evaluated the diagnostic performance of a novel multiplex ELISA to detect anti-*M. bovis* IgG in bovine nasopharyngeal swabs. In cattle, secretory IgG in the nasal and upper respiratory tract is as important as or more than sIgA (19, 20). Our findings showed that this assay provides a highly accurate and reproducible method for profiling localized antibody responses without the need for invasive sampling.

A different performance among the three targeted antigens was observed in this study. High accuracy was obtained with both K-0310 and K-0320, whereas the MilA antigen consistently underperformed. MilA exhibited instances of spot variance exceeding tolerance limits, demonstrated poor predictive accuracy (51.5%) when evaluated against *M. bovis* DNA status, and was ultimately non-informative in the LCA model. The failure of MilA in this context suggests it may not be a suitable target for mucosal IgG detection in the bovine respiratory tract, potentially due to a lack of strong, localized immunogenicity compared to K-0310 and K-0320. However, MilA has been documented as a major *M. bovis* antigen (19, 21). MilA is a large, surface-exposed membrane lipoprotein with lipase, lipid-binding, and glycosaminoglycan-binding activities; antibodies against its C-terminal domain inhibit *M. bovis* growth *in vitro*, suggesting that MilA contributes to nutrient acquisition and metabolic fitness required for virulence *in vivo* (21, 22). We hypothesize that anti-MilA IgG levels may increase during BRD, improving MilA performance. Therefore, assessing MilA performance in nasopharyngeal swabs from beef cattle with BRD is warranted. As for K-0310, a transposon mutant with disruption of this gene (MBOVPG45_0310) was identified in a genome-wide mutant library, and orthologs of K-0310 are conserved among several pathogenic mycoplasmas, suggesting an important role in nucleotide scavenging (23). However, direct virulence phenotyping of K-0310 or the neighboring K-0320 (MBOVPG45_0320) genes has not yet been reported.

One of the most significant challenges in validating novel mucosal antibody tests is the lack of a true “gold standard” (19, 20, 24). Initially, we utilized the presence of *M. bovis* DNA, determined via digital qPCR, as the ground truth. Under this framework, the best-performing custom cutoffs (K-0310 and K-0320 evaluated in series) yielded a high specificity of 88% but a remarkably low sensitivity of 30%. This discrepancy highlights a fundamental biological reality: the physical presence of pathogen DNA (indicating current colonization or infection) does not perfectly correlate with the presence of target-specific IgG, which represents a past or ongoing adaptive immune response. Cattle may harbor *M. bovis* DNA acutely before mounting a detectable IgG response, or conversely, they may have cleared the bacterial DNA while maintaining high levels of mucosal antibodies (13, 25).

To overcome the limitations of this imperfect reference standard, we employed an LCA, which is a powerful statistical tool that estimates unobserved subpopulations (the true *M. bovis* IgG-positive and negative animals) based on the observed test results (16). The rationale for selecting this statistical tool is that we expect two subpopulations of samples, those with and without *M. bovis* IgG. However, in the absence of a gold standard that can neatly define the two subpopulations, LCA allows for estimating them from the findings of the test being evaluated. The LCA model estimated an IgG positivity prevalence of 17.1% in our samples. Using these LCA-derived classifications, the assay's diagnostic validity improved substantially. When K-0310 and K-0320 were interpreted in parallel using the LCA-based cutoffs, the assay achieved an accuracy of 97.59%, a sensitivity of 100%, and a specificity of 95.18%. These values are similar to those reported by Wawegama et al. (22, 26) and Salgadu et al. (27), however, these assays were based on the same recombinant MilA protein that was found to be poorly predictive in this study. This may be due to cross-reactivity with other mycoplasmas in the upper respiratory tract, such as *M. agalactiae*, which possess a similar protein to *M. bovis* MilA (28, 29). Further research is needed to elucidate the effect of cross-reactivity with other mycoplasmas on the results of these assays.

In addition to diagnostic accuracy, the assay demonstrated robust operational reliability. Reproducibility assessments revealed generally very low variability between different operators and independent analytical runs. Although systematic differences were observed, these variations did not compromise clinical interpretation. When applying the established cutoffs, inter-operator agreement was very high. Furthermore, it was shown that the assay can reliably identify positive samples at a 1:2 dilution factor, meeting the 95% target probability threshold for both cutoff schemes.

Although these results are highly promising, certain limitations must be acknowledged. The variable wash volume recovered from the nasopharyngeal swabs warrants stringent sample normalization or a tolerance for dilution within the assay, which our analytical sensitivity results suggest is currently adequate up to a 1:2 dilution. Additionally, the samples evaluated were sourced from a specific cohort of healthy crossbreed feedlot steers, meaning further validation may be required to generalize these cutoffs to other groups, such as young dairy calves, cow-calf operations, or feedlot cattle with BRD.

## Conclusions

In this proof-of-concept study, we validated a novel multiplex ELISA platform for the detection of anti-*M. bovis* IgG in bovine nasopharyngeal swabs, providing evidence that K-0310 and K-0320 are highly effective target antigens while demonstrating the inadequacy of MilA in this specific sample matrix. Importantly, the application of Latent Class Analysis showed that evaluating K-0310 and K-0320 in parallel provides excellent diagnostic accuracy (97.59%), mitigating the limitations of using concurrent pathogen DNA presence as an imperfect gold standard. With high inter-operator reproducibility, this assay, when applied to nasopharyngeal swabs, has the potential to become a valuable, minimally invasive tool for profiling localized mucosal humoral immunity in beef cattle.

## Data Availability

The raw data supporting the conclusions of this article will be made available by the authors, without undue reservation.
