# Supplementary material for: Detection of anti-Mycoplasma bovis IgG in bovine nasopharyngeal swabs
Source: Front Vet Sci. 2026 Jun 11;13:1856081. doi: 10.3389/fvets.2026.1856081 (PMC13295102; doi:10.3389/fvets.2026.1856081)
Supplement: Supplementary file 1 [file Data_Sheet_1.docx]

Supplementary Material

- TaqMan Primer/Probe Sets
  - *Mycoplasma bovis*
    - Forward: TCAAGGAACCCCACCAGAT
    - Reverse: AGGCAAAGTCATTTCTAGGTGCAA
    - Probe : 6FAM-TGGCAAACTTACCTATCGGTGACCCT-QSY
  - *Mannheimia haemolytica*
    - Forward: GCCGTTGTTTCAACCGCTAAC
    - Reverse: CGTGTTCCCAAACGTCTAAGAC
    - Probe : ABY-TCGGATAGCCTGAAACGCCTGCCAC-QSY
  - *Histophilus somni*
    - Forward: GCAATGATGTACCWGCCAAAG
    - Reverse: CCTTCAGCTCACCATTACCATA
    - Probe : VIC-TTGCTTACGTCCAAACCGTCGTGT-QSY
  - *Pasteurella multocida*
    - Forward: ATCCCTGCGTTACAGAGTTTAG
    - Reverse: GACGYGGGYAGTACCATAAA
    - Probe : JUN-TTGATGCCTTCTTTGCGGGTTTCG-QSY
- Positive control : DNA from *Mycoplasma bovis* ATCC 25523, *Mannheimia haemolytica* ATCC 33396, *Pasteurella multocida* ATCC 43137, and *Histophilus somni* ATCC 700023.

**Supplementary Figure 1.** Receiver Operating Characteristic (ROC) Curve for *Mycoplasma bovis* DNA-based cutoffs. The cutoffs correspond to average median intensity ≥ 18.75 for K-0310 and ≥17.5 for K-0320.

**Supplementary Figure 2.** Bland-Altman plot for MilA. The average of MilA intensity values between both operators is plotted against the difference in MilA values between operators. The blue horizontal line is the average agreement and the red horizontal lines are the 95% lower and upper limits.

**Supplementary Figure 3.** Limit of detection using a *Mycoplasma bovis* DNA-based cutoff. The y axis represents the probability of detection using *Mycoplasma bovis* DNA-based cutoff and the x axis depicts the dilution factor (transformed as log_2_). Vertical lines represent 95% CI adjusted for clustering. The horizontal dashed line marks the target probability of 0.95.
